# A Substitute for Human Milk

**Published:** 1866

**Authors:** 


### A Substitute for Human Milk.

We have received a sample of an article prepared under the personal supervision of W. H. Peabody, chemist and pharmaceutist, assisted by Prof. George Hadley, designed to be a substitute for the natural food of infants. It is prepared in such manner as to add to the milk of the cow the chemical elements and properties of human milk, and thus to furnish food for infants similar in taste, appearance and chemical composition to healthy human breast milk. This article has borne the test of trial, and we have the testimony of many of our most experienced and discriminating physicians, that it agrees in most cases with infants much better than any other article yet introduced to supply the place of breast milk. Some of these physicians have not only prescribed it to patients, but have also used it in their own families, and speak of it in terms of unqualified approval.

In all animals belonging to the great class of *mammals* the sole nourishment of the young is milk, which contains in itself three classes of elements whose simultaneous use is essential to nutrition, viz: the mineral, the oleaginous and sacherine, and the albuminous. Different animals, however, require these, to be supplied in different proportions, and as one grown up animal lives on one kind of food, and another on a very different, so the milk supplied to the young varies much in the relative proportions of its three great constituents. For example: human milk contains more of the second and less of the third class of elements than the milk of the cow; it is also more bluish, has a sweeter taste, turns sour less readily, and forms a gelatinous curd which is not so dense as that of the the cow's milk, and which is more easily digested. The elements necessary to convert cow's milk into human milk, so far as chemical composition is concerned, has no doubt been very perfectly supplied in this compound prepared by Mr. Peabody, Prof. Hadley's approval being sufficient guarantee of its genuineness and chemical purity. Its practical value is already established with those who have made trial of its virtues, and it will be presented to the public, through the profession, with confidence that it will answer every reasonable expectation.

